# Physicochemical Properties and Bioaccessibility of Phenolic Compounds of Dietary Fibre Concentrates from Vegetable By-Products

**DOI:** 10.3390/foods11172578

**Published:** 2022-08-25

**Authors:** Ana A. Vaz, Isabel Odriozola-Serrano, Gemma Oms-Oliu, Olga Martín-Belloso

**Affiliations:** Department of Food Technology, University of Lleida—Agrotecnio CERCA Center, Av. Alcalde Rovira Roure191, 25198 Lleida, Spain

**Keywords:** fibre, artichoke, red pepper, cucumber, carrot, phenolic compounds, in vitro gastrointestinal digestion, in vitro colonic digestion, bioaccessibility

## Abstract

The agro-food industry generates a large volume of by-products, whose revaluation is essential for the circular economy. From these by-products, dietary fibre concentrates (DFCs) can be obtained. Therefore, the objective of this study was to characterise (a) the proximal composition by analysing soluble, insoluble and total Dietary Fibre (DF), (b) the physicochemical properties, and (c) the phenolic profile of artichoke, red pepper, carrot, and cucumber DFCs. In addition, the bioaccessibility of phenolic compounds was also evaluated after in vitro gastrointestinal and colonic digestions. The results showed that the DFCs had more than 30 g/100 g dw. The water holding and retention capacity of the DFCs ranges from 9.4 to 18.7 g of water/g. Artichoke DFC presented high concentration of phenolic compounds (8340.7 mg/kg) compared to the red pepper (304.4 mg/kg), carrot (217.4 mg/kg) and cucumber DFCs (195.7 mg/kg). During in vitro gastrointestinal digestion, soluble phenolic compounds were released from the food matrix, chlorogenic acid, the principal compound in artichoke and carrot DFCs, and hesperetin-7-rutinoside in red pepper cucumber DFCs. Total phenolic content decreased after in vitro colonic digestion hence the chemical transformation of the phenolic compounds by gut microbiota. Based on the results, DFCs could be good functional ingredients to develop DF-enriched food, reducing food waste.

## 1. Introduction

Dietary fibre (DF) is the edible part of vegetables that is neither digested by human enzymes nor absorbed in the small intestine, leading to a fermentation process by gut microbiota in the large intestine [1]. DF can be classified as (a) soluble dietary fibre (SF), referred to as the DF fraction soluble in water at 100 °C and pH 6–7, being pectin, gums, inulin mucilage, and β-glucans, the main components; and (b) insoluble dietary fibre (IF), the non-soluble fraction composed of hemicellulose, cellulose, and lignin [2,3].

Along with DF solubility and physicochemical characteristics, its fractions, IF and SF, have diverse physiological properties [4]. On the one hand, IF mainly (a) increases the faecal bulk owing to porosity and low density, (b) promotes intestinal mobility, and (c) increases the frequency of defaecation, decreasing the transit time, and (d) traps toxins [5,6]. On the other hand, SF is more (a) susceptible to fermentation by colonic bacteria, modulating the gut microbiota and producing short-chain fatty acids; (b) regulates satiety; (c) forms gels that decrease blood glucose levels and attenuate cholesterol levels in plasma, and (d) increases viscosity reducing glycemic response and decreasing absorption of cholesterol and free fatty acids [5,7,8]. Therefore, due to their specific physiological properties, each fraction of DF, SF, and IF complement each other [9]. Apart from DF solubility, other physicochemical characteristics of DF play a role in gut health. WHC, WRC, and the swelling capacity of DF create an aqueous phase that increases the total volume of the intestinal content. The large intestine dilutes nutrients, slowing down their absorption. In the large intestine, gut microbiota can quickly enter the aqueous phase to break down the structure of DF. In addition, OHC and ORC properties are related to the absorption of fat by the DF, reducing calorie intake, and regulating cholesterol absorption [10,11].

Despite the beneficial effects of DF, the current per-capita intake is lower than recommended per day [12]. WHO and FAO guidelines recommend a daily intake of >25 g of DF obtained predominantly through the consumption of 400 g of fruits and vegetables [13]. Furthermore, low DF intake causes diseases such as obesity, diabetes mellitus, inflammatory bowel diseases [14], several types of cancer [15,16], constipation [17], and diverticulosis [18]. The food industry is working to develop new DF enriched foods to prevent low DF intake-related diseases by increasing DF consumption. For that purpose, the food industry is looking for new sources of DF. To achieve this goal, Dietary Fibre Concentrates (DFCs), obtained from the processing of vegetables, could be considered as novel food ingredients [2,19].

The agro-food industry generates waste (pods, leaves, seeds, husks, roots, etc.) after processing raw vegetables such as artichoke, red pepper, cucumber, and carrot. That waste reaches one-third of the total food produced for human consumption [20], causing environmental issues [21]. Therefore, a solution to the problem could be introducing these products in foods, reducing garbage and pollution, and the concepts of sustainable diets and the circular economy would be encouraged [22]. Moreover, these parts of the plant, without any potential value, are rich in DF [23] as well as other compounds such as minerals, vitamins [24], and phenolic compounds [25]. This way, functional food enriched in DF and bioactive compounds could be developed [26].

Phenolic compounds are a heterogeneous group with different structures, solubility, and polarity. Phenolic compounds are found inside the vacuole of the plant cell and bound to the vegetal cell wall, interacting with other molecules as macronutrients such as proteins, digestible carbohydrates, lipids, and DF that protect phenolic compounds during gastrointestinal digestion from oxidation. Non-covalent bonds link phenolic compounds to digestible carbohydrates and lipids. Non-covalent and covalent bonds link proteins [27,28].

These interactions between phenolic compounds and macronutrients decreased the bioaccessibility of phenolic compounds and, consequently, bioavailability. However, the interactions between phenolic compounds and the food matrix change during digestion due to the food undergoing several different transitions and transformations. In the stomach and small intestine, acid pH and enzymes can break down these interactions among proteins, digestible carbohydrates, lipids, and phenolic compounds. In the large intestine, the enzymes of the gut microbiota can break the DF matrix, releasing phenolic compounds from the vacuole and the cell wall, where phenolic compounds interact with molecules like pectin. Once these modifications occur, phenolic compounds are released from the food matrix, becoming bioaccessible along the intestinal tract to reach their target organ [27,29]. Once phenolic compounds are in their target organ, they can perform physiological functions, protecting the body from oxidative stress-related diseases [30] since phenolic compounds show high antioxidant activity [31].

This study aims to characterise DFCs of artichoke, carrot, red pepper, and cucumber obtained from agro-food industry wastes in terms of DF fractions, centesimal composition, and physicochemical properties. In addition, phenolic compounds profile and bioaccessibility after gastrointestinal and colonic digestion of the DFCs will be evaluated.

## 2. Materials and Methods

### 2.1. Materials

Vegetable DFCs (artichoke, red pepper, cucumber, and carrot) were kindly supplied by Indulleida S.A. (Lleida, Spain). All were the result of drying the bagasse (pulp and skin) obtained after pressing vegetables for their juice extraction process.

### 2.2. Methods

#### 2.2.1. Dietary Fibre Concentrates Characterization

##### Dietary Fibre Fractions and Proximate Composition

TDF, SF, and IF content was determined by the AOAC 991.43 gravimetric method [32]. This method is based on the enzymatic removal of proteins with proteases from DFC and the separation of soluble and insoluble fractions by centrifugation during 20 min at 10,000× *g*. The results are reported as g/100 g dry weight (dw) of DF. The moisture, protein, fat, ash, and fibre contents of each by-product were determined using Association of Official Analytical Chemists (AOAC) methods [33]. The moisture content was determined by heating 0.2 g of each sample in an oven (Oven 2010, JP Selecta S.A., Barcelona, Spain) at 105 °C until a constant weight was achieved. The total protein content was estimated by quantifying nitrogen after digestion with sulphuric acid using a Kjeldahl distillatory (Bloc Digest6, JP Selecta, Barcelona, Spain). Fat was obtained by extracting 2 g of each by-product in a Soxhlet using hexane as the solvent extractor solution. Ash was determined gravimetrically using a muffle furnace device (Carbolite CWF 1100, Carbolite Gero Ltd., Hope, UK) at 550 °C for 24 h. Digestible carbohydrates were calculated as the difference between 100 and the total value of protein, fat, and ash.

##### Physicochemical Properties

pH and Acidity

The pH was evaluated potentiometrically with a pH-meter (Crisol 2001; Crison Instruments S.A. Alella, Barcelona, Spain) mixing 10 g of DFC with 100 mL of distilled water. The acidity was measured thorugh the titration of these solutions with NaOH (0.1 N) until reaching a of pH 8.1. Results were expressed as citric acid g per 100 mL of solution for the DFC.

Apparent Density

The apparent density was calculated as the DFC weight poured to fill a 10 mL graduated cylinder. Results were expressed as weight (g) per unit volume (mL).

Solubility

Solubility analyses were performed according to Robertson et al. [34]. Briefly, DFCs were mixed with distilled water (1:10 *w*/*v*) and stirred for 3 h at room temperature. Then, the mixture was centrifuged, and the pellet was dried overnight at 60 °C. Solubility was expressed as the percentage of DFC weight losses after the procedure.

Swelling Capacity

A 10 mL graduated cylinder was filled with 1 g of each DFC and 10 mL of water. Then, the mixture was combined and kept for 24 h at room temperature. Swelling capacity was expressed as the volume occupied by the hydrated DFC divided by the initial weight [34].

Water (WHC) and Oil (OHC) Holding Capacity

The WHC and OHC of the DFC were determined by mixing 2 g of sample with 15 mL of water and soybean oil, respectively, for 24 h. Results were expressed as the amount of water or oil held by DFC weight (g) [35].

Water (WRC) and Oil (ORC) Retention Capacity

Water and oil retention capacity were measured after centrifugation (3000× *g* for 20 min) of the DFC mixed with water or soybean oil by the difference from the initial weight. Results were expressed as the amount of water or oil retained by DFC weight (g) [35].

#### 2.2.2. Digestion of DFC

##### In Vitro Gastrointestinal Digestion

A portion of 5 g of each DFC were digested in vitro following the INFOGEST [36] protocol. Briefly, for the stomach phase, each DFC was mixed with simulated gastric fluids (SGF) containing pepsin (2000 U/mL in the final mixture) (Sigma-Aldrich, Madrid, Spain), CaCl_2_ (H_2_0) (0.3 M), Milli-Q water and HCl (6 M) and incubated (Incubator OPAQ, OVAN, Barcelona, Spain) for 2 h at 37 °C at 100 rpm. For the intestinal phase, the contents from the stomach phase were placed in a water bath (37 °C), adding simulated intestinal fluids (SIF) (0.150 M NaCl and 0.01 M CaCl_2_), bile extract (54 mg/mL), and pancreatin (75 mg/mL) (Sigma-Aldrich, Madrid, Spain). To maintain a pH of 7, 0.25 M NaOH was added constantly for 2 h using a pH-stat (Metrohm USA Inc., Riverview, FL, USA). Finally, content from the intestinal digestion was centrifuged for 50 min at 14,000 rpm, considering the supernatants as the absorbed portion and the pellets as the unabsorbed portion. The supernatants were stored at −40 °C, and the pellets were lyophilized for 48 h for further experiments.

##### In Vitro Colonic Digestion

Faecal samples were collected from five healthy donors who had not taken antibiotics within the last three months. The study was approved by the Ethics Committee of the Hospital Universitari Arnau de Vilanova, Lleida, Spain, CEIC-1980. Donors transported their faeces to the laboratory in sterile pots, which were processed within the following 3 h after defecation. Faecal samples were mixed (10%) with a fermentation medium prepared according to Durand et al. as follows [37]: 9.240 NaHCO_3_, 3.542 Na_2_HPO_4_·2H_2_O, 0.470 NaCl, 0.450 KCl, 0.227 Na_2_SO_4_·10H_2_O, 0.055 CaCl_2_ (anhydrous), 0.100 MgCl_2_·6H_2_O, and 0.400 urea with 10 mL of added trace element solution containing 3680 FeSO_4_·7H_2_O, 1159 MnSO_4_·H_2_O, 440 ZnSO_4_·7H_2_O, 120 CoCl_2_·6H_2_O, 98 CuSO_4_·5H_2_O, 17.4 Mo_7_(NH_4_)_6_O_24_·4H_2_O in milligrams per litter. The fermentation medium was adjusted to a pH of 7.0 using HCl containing glycerol at 15% and stored at −80 °C for further analysis.

The in vitro colonic digestion was performed with the 1:10 (*v*/*v*) faecal slurry, the fermentation medium, and 1% (*w*/*v*) of the unabsorbed portion from the in vitro gastrointestinal digestion of artichoke, red pepper, cucumber, and carrot DFCs, and incubated at 37 °C for 48 h in anaerobic conditions (BioMérieux^®^ S.A., Marcy-l’Étoile, France).

#### 2.2.3. Phenolic Profile

A portion of vegetable DFCs, lyophilized from the unabsorbed fraction in the intestine, the absorbed fraction in the small intestine, and the in vitro colonic fermentation of the DFCs, were homogenized with 70% ethanol in Milli-Q water and homogenised at 1600 rpm for 2 min with an Ultra-Turrax T25-Basic mixer. An ultrasonic processor (Hielscher Ultrasonic Processor GmbH, mod UP4000S, Teltow, Germany) with a Titanium tip H4 was used at 24 kHz with a nominal amplitude of 125 µm for 120 to optimize the phenolic extraction. Then, the mixture was centrifuged at 12,500 rpm at 4 °C for 15 min, and the extract was passed through a 0.45 μm filter. The resulting extracts were stored at −18 °C in darkness until further analysis. The detection and quantification of the individual phenolic compounds were performed by UPLC-MS/MS on an AcQuity Ultra-Performance^TM^ liquid chromatography/tandem mass spectrometry equipment (Waters, Milford, MA, USA) working at the chromatographic conditions described by Yuste et al. [38]. Individual phenolic compounds quantification was carried out by comparison with calibration curves of commercial standards.

## 3. Results and Discussion

### 3.1. Dietary Fibre Fractions and Proximate Composition

The artichoke, cucumber, and red pepper DFCs presented over 30% of Total DF (TDF). Artichoke DFC had the highest content with 54.7 g/100 g dw of TDF, followed by red pepper DFC with 43 g/100 g dw. Non-significant differences in TDF were observed between carrot (33.8 g/100 g dw) and cucumber DFCs (33.7 g/100 g dw) (Table 1). Several studies carried out in fresh whole vegetables reported different concentrations of TDF compared to the DFCs considered in the present study. For instance, Uthpala et al. [39] found that TDF in fresh cucumber varied from 0.7 to 20.4%. In raw red pepper, Vega-Gálvez et al. [40] reported 1.2 g/100 g of TDF of fresh weight. Regarding the amount of TDF in DFCs, Grigelmo-Miguel & Martín-Belloso [26] reported 58.8 g/100 g dw of TDF for artichoke DFC, and Mármol et al. [41] reported values of 22.48 g/100 g dw of TDF for cucumber DFC and 19.95 g/100 g dw of TDF for red pepper DFCs. Therefore, there is no clear relationship in TDF concentration between fresh products and DFCs, and between the TDF reported for DFCs. The different distribution of DF in the parts of the plant can explain these discrepancies. Indeed, the carrot’s skin presented a higher percentage of DF than the phloem and xylem [42]. Artichoke outer bracts had a greater concentration of DF than internal parts [43]. In red pepper and cucumber, the pomace contains less percentage of DF than the peel [44]. These differences among parts of the vegetable can explain the higher concentrations of TDF in analysed DFCs compared with the studies cited above.

**Figure 1:**
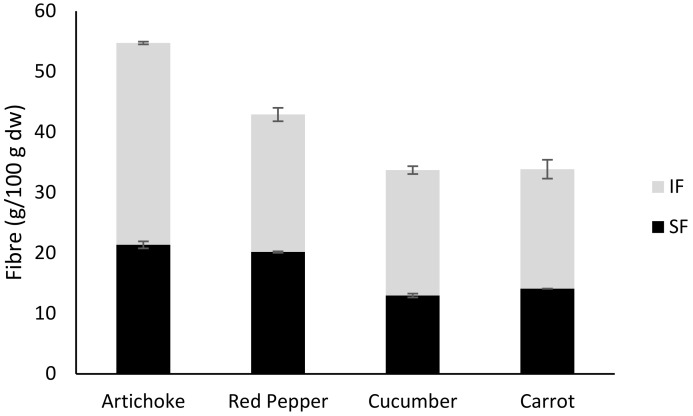
Total, soluble (SF) and insoluble (IF) dietary fibre (DF) content of artichoke, red pepper, cucumber, and carrot dietary fibre concentrates. SF: soluble dietary fibre and IF: insoluble dietary fibre.

IF’s content is slightly higher than that of SF in all DFCs studied, ranging from 53% to 61% of IF and from 38% to 47% of SF (Figure 1). According to other studies, the IF fraction is higher than the SF fraction in vegetable by-products [26,45,46]. As mentioned above, IF and SF complements each other in their functional properties. It has been reported that a good balance between IF and SF would be 70–50% of IF and 30–50% of SF [47]. A significantly higher percentage of SF has been observed in the studied DFCs compared to other vegetable by-products published in the existing literature. For instance, Grigelmo-Miguel & Martín-Belloso [26] reported that IF values of vegetable DFCs ranged from 21.2% to 24.3% and SF from 75.7% to 78.8%. Quintero et al. [48] characterized IF and SF of non-edible bracts of artichoke, reporting 82.5–55.2% of IF. Clementz et al. [49] found that by-products from carrots had 72.83% of IF and 27 SF. Moreover, greens had a major portion of SF than cereals [26]. Therefore, the analysed DFCs are good sources of IF and SF, making them good candidates to add into foods with the purpose of developing DF enriched foods.

Carrot has the highest percentage of digestible carbohydrates, with more than 50 g/100 g dw. Cucumber, red pepper, and artichoke DFCs had less than 40 g/100 g dw (Table 1). Previous studies reported less than 20 g/100 g of carbohydrates in carrot pomace [50]. This might be due to differences in maturity during the stored time since the stress caused by pre-harvest and post-harvest adaptation changes carbohydrate concentrations [51]. Digestible carbohydrates are the macronutrient responsible for providing energy to the human body since the human metabolism can easily break down digestible carbohydrates into glucose. Glucose is the main source of energy for cells, tissues, and organs. Moreover, glucose is the substrate of metabolic pathways such as glycolysis and glycogenesis [5].

The protein content was more than 10 g/100 g dw in cucumber, red pepper, and artichoke DFCs and less than 10 g/100 g dw in carrot DFCs (Table 1). Previous studies have reported similar values for cucumber, red pepper, and artichoke DFC [26,41]. According to EU regulations, artichoke, red pepper, and cucumber DFCs are a good source of protein as they have more than 12% protein of the total content. Fat only represented less than 2 g/100 g dw for artichoke, cucumber, and carrot DFCs and 4.5 g/100 g dw for red pepper. Likewise, the EU regulation determined that artichoke, cucumber, and carrot DFC are low in fat, as the solid product contains no more than 3 g of fat per 100 g. Currently, scientific research is focused on developing functional foods that are a source of protein also low in fat as a meat alternative [52]. For this objective, artichoke and cucumber DFCs could be good candidates due to their characteristics.

Ash content indicates the inorganic part of the DFC. The studied DFCs showed ash contents between 4.7 and 7.1 g/100 g dw (Table 1), which were similar to those determined by Grigelmo-Miguel & Martín-Belloso [26] and Mármol et al. [41] in red pepper, cucumber and artichoke DFCs.

The selected vegetable DFCs have a low moisture content (≤10 g/100 g fresh weigh), as can be seen in Table 1. Low concentrations of water increases storage time, slowing fungi growth and chemical reactions [53].

### 3.2. Physicochemical Properties

Table 2 shows the physicochemical properties of the selected vegetable DFCs. Their pH values ranged from 4 to 5. Within this range, the values were statistically different for all DFCs, showing the cucumber DFC with the lowest pH value and the highest acidity (0.23 g acid/100 mL). Despite the natural acidity of the DFCs, their addition to new foods could not change the pH of the resulting product, which might therefore facilitate the incorporation of DFCs into foods without changing their acid-basic balance. In fact, Grigelmo-Miguel and Martin-Belloso [54] demonstrated no change in pH in the final product when a fruit DFC with acidic pH was added to the jam.

Apparent density depends on the structural characteristics and the particle size of the DFC [2]. Lower apparent densities (less than 0.40 g/mL) were determined in artichoke, cucumber, and red pepper DFCs than in carrot DFC (0.47 g/mL) (Table 2). Low apparent density suggests great surface area and polar groups, which can be related to better binding of water and lipids, restricting water and lipid flux, and increasing the swelling capacity (SC) [35,50].

Differences in SC among vegetable DFCs appear to be related with t their nutritional components [55]. SC of the analysed DFCs varied from 8.04 mL/g to 11.10 mL/g (Table 2), in concordance with He et al. [10], who reported that SC of vegetable by-products varied from 5.35 to 18.7 mL/g.

WHC and WRC are DF hydration properties. WRC determines the water retained by the food matrix after a stress situation caused by an external force, such as centrifugation [56], measuring physically bound water in the food matrix [45]. On the other hand, WHC measures the DFC-associated water without applying any external force to separate water molecules from the food matrix. For this reason, higher WRC than WHC values were obtained. Moreover, WHC and WRC results of analysed DFCs were directly linked to each other, and both increased or decreased simultaneously. Red pepper DFC had the highest WHC (18.7 g water/g) and WRC (15.5 g water/g), followed by artichoke (WHC 11.0 g water/g, WRC 10.5 g water/g) (Table 2). These values indicated that red pepper DFC could capture hydrophobic molecules in a more efficient way. This led to changes in physicochemical properties and physiological behaviour. Regarding physicochemical properties, when a DFC is added to a new food, elevated WHC and WRC values can change the texture and viscosity of the final product changing rheological properties [23,57]. As for physiological behaviour an elevated WHC and WRC help to increase faecal bulk [8], particularly increasing the aqueous phase and micelles [58].

In the same way, the higher the OHC was, the greater the ORC and vice versa. Moreover, lower OHC than ORC values were obtained for each DFC. This could be attributed to the cleavages between oil and food matrix since weakly bound oil molecules can be separated from the food matrix by centrifugation (ORC). But when no external force was applied, oil and DFC remained linked regardless of the strength of the interaction (OHC). Artichoke DFC presented the highest OHC (2.0 g oil/g) and ORC (1.9 g oil/g). A low value was reported by López et al. [59] (1.31 g oil/g) for the artichoke by-product. Lower values were found for red pepper, cucumber, and carrot DFCs (≤1.5 g oil/g for ORC and ORC) than artichoke DFC.OHC and ORC are directly related to physiological and physicochemical properties. These results indicated that the artichoke DFC could stabilise high-fat products and emulsions [2]. From a physiological point of view, artichoke DFC could retain fat during digestion reducing fat absorption and attenuating in plasma the levels of total cholesterol [60].

### 3.3. Phenolic Compounds

Total and individual phenolic compounds of vegetable DFC are shown in Table 3. Artichoke DFC presented high concentration of total phenolic compounds (8340.7 mg/kg) compared to the red pepper (304.4 mg/kg), carrot (217.4 mg/kg) and cucumber DFC (195.7 mg/kg). In literature, important differences in terms of the content of total phenolic compounds have been reported between whole vegetable and vegetable by-products. Regarding artichoke, Jiménez-Moreno et al. [61] reported a concentration of phenolic compounds of 2558 mg/kg in artichoke waste, mainly composed of external bracts, leaves, and stems; however, higher amounts (69,979 mg/kg) were found in the whole artichoke by Domínguez-Fernández et al. [62]. These differences suggested that artichoke heads could be richer in phenolic compounds than other parts. On the contrary, the total phenolic content of whole carrots ranged from 159 to 259 mg/kg depending on the cultivar [63], but in carrot peels, was approximately 13,800 mg/kg [64]. The phenolic amount of vegetables depends not only on factors such as variety, climate, and harvest time but also on the considered edible parts since phenolic compounds are intrinsically interacting mainly with DF [65]. In this way, the high number of phenolic compounds in artichoke DFC could be related to the fact that these DFCs presented the highest amount of TDF (Table 1).

Phenolic acids represented more than 98% of PC in artichoke and carrot DFC. The main phenolic acids in artichoke DFC were hydroxycinnamic acids, especially 3,5-O-dicaffeoylquinic acid (5478.6 mg/kg), chlorogenic acid (51,801.6 mg/kg), and caffeic acid (551.5 mg/kg), accounting for the 95% of the phenolic acids. In carrot DFC, just chlorogenic acid represented more than 82% of the total phenolic acids. In addition, the dihydroxybenzoic acid, protocatechuic acid, was detected in high amounts in artichoke (320.5 mg/kg) and carrot (10.9 mg/kg) in comparison to other phenolic acids. According to our results, Domínguez-Fernández et al. [62] and Zhang and Hamauzu [64] reported that hydroxycinnamic acids are the most representative phenolic compounds linked to DF in artichoke and carrot vegetables. On the other hand, the most abundant flavonoids in artichoke DFC were luteolin-7-O-glucoside and luteolin, with very low amounts of the rest of the flavonoids. These results agree with those reported by Jiménez-Moreno et al. [61] since luteolin derivatives are much more abundant in artichoke by-products such as leaves and steam than in the edible part of this vegetal, where the most important flavonoid would seem to be the apigenin derivatives.

The most representative PC in cucumber and red pepper DFC were flavanones, with a concentration of 149.9 mg/kg and 145.6 mg/kg, respectively. Specifically, hesperetin-7-rutinoside was the main flavanone in both DFC, accounting for more than 70% of the total flavanone. In addition, eriodictyol-7-O-rutinoside was also abundant in red pepper. Flavonol compounds were also identified in high proportions in red pepper DFC, with quercetin-3-L-rhamnoside as the most representative compound accounting for 94% of the amount of this group. Although there is some literature reporting the concentration of TPC in whole red pepper and cucumber by spectrophotometric techniques, no previous studies have been found analysing PC in neither whole nor by-products from these vegetables by HPLC-MS chromatographic methodology.

### 3.4. Bioaccessibility of Phenolic Compounds

The effect of gastrointestinal and colonic digestions on the phenolic profile of the selected DFCs can be seen in Table 4. Three fractions were analysed after the in vitro digestion of the DFC. Firstly, after gastrointestinal digestion, phenolic compounds were determined in two different fractions being (a) the intestinal bioaccessible fraction, which was soluble and, therefore, the fraction potentially absorbed in the small intestine, and (b) the non-bioaccessible fraction, which was insoluble in the intestine that had undergone in vitro colonic digestion. Secondly, the colonic bioaccessible fractions were obtained after the in vitro colonic digestion of the intestinal non-bioaccessible fraction. As can be seen in Table 4, the concentration of bioaccessible phenolic compounds was between 2-folds (red pepper and cucumber DFCs) to 13-folds (carrot DFC) enhanced after gastrointestinal digestion compared to the content in the undigested fractions. This finding could be partially explained by the breakdown of the linkages between phenolic compounds and macronutrients and DF during digestion. Phenolic compounds have the ability to interact not only with DF by also with other macronutrients present in foods such as carbohydrates, proteins, or lipids [27]. Therefore, phenolic compounds might be released from the food matrix in the upper area of the gastrointestinal tract by the action of digestive enzymes and physiological fluids. High acid conditions during the stomach phase might cleavage some covalent bounds of phenolic compounds and facilitate the release from the food matrix [66]. Interestingly, the compound mainly released in the intestinal phase in artichoke and carrot DFCs was chlorogenic acid, accounting for about 75% of the total bioaccessible compounds in the intestinal phase. The flavanone, hesperetin-7-rutinoside, was the phenolic compound with the highest concentration in soluble intestinal fractions of digested cucumber DFC, representing about 77% of the soluble phenolic compounds. In the case of red pepper DFC, the main phenolic compounds that solubilize in the intestinal phase were the flavonoids hesperetin-7-rutinoside, quercetin-3-L-rhamnoside, and quercetin, as well as some phenolic compounds such as ferulic acid and protocatechuic.

Analysing the concentration of individual phenolic compounds, the intestinal bioaccessible fraction of each phenolic compound was similar to or higher than those quantified in the undigested DFCs with the exception of salicylic acid, luteolin-7-O-glucoside, and luteolin, which were only detected in the non-bioaccessible intestinal fraction. Thus, it can be hypothesized that these three phenolic compounds are not capable of being extracted by intestinal fluids. In addition, a high proportion of phenolic acids and flavonoids (240–9678 mg/kg) were still detected in the non-bioaccessible fraction, indicating that these compounds are bounds to the matrix, so they will reach the colon and will be used as substrates by the gut microbiota. Naringenin-7-O-rutinoside was the unique phenolic compound that was not detected in intestinal insoluble or soluble colonic fractions after the gastrointestinal digestion of DFCs, indicating that this flavanone is potentially absorbed in the small intestine [67].

Total phenolic concentration sharply decreased (>75%) in all the DFCs after colonic digestion compared to phenolic compounds present in insoluble intestinal fraction. Flavonols, flavanones, and flavones were not detected in the soluble colonic fraction, meaning that these compounds have been transformed into other phenolic metabolites by the microorganisms present in the gut. Small amounts of chlorogenic acid and 3,5 dicaffeoyl quinic acid were bioaccessible after the colonic digestion of the considered DFCs. In addition, the release of some phenolic acids such as o-salicylic acid, vanillic acid, and syringic acid after the colonic digestion step exceeded that found in the intestinal non-bioaccessible fraction, which suggests that these phenolic acids were completely released from the matrix or could be metabolized from more complex polyphenols. On the other hand, luteolin, luteolin-7-O-glucoside, and luteolin-8-C-glucoside presented in the intestinal insoluble fraction could not be quantified after colonic digestion because they were degraded, or the contents were lowered to the limit of quantification. The low amounts of phenolic compounds after colonic digestion can be related to the fact that when phenolic compounds reach the colon, they undergo chemical alterations by the action of gut microbiota [68]. In fact, several chemical reactions have been previously reported, such as phenolic deglycosylation, dehydroxylation, demethylation, deconjugation, epimerisation, ring cleavage, hydrolysis, and chain-shortening [69].

## 4. Conclusions

Based on the observed results, the analysed artichoke, cucumber, red pepper, and carrot DFCs from vegetable by-products can be used as functional ingredients for developing DF-enriched food. DFCs can also provide bioaccessible phenolic compounds to consumers. Moreover, a source of protein and low in fat novel foods could be obtained by adding artichoke and cucumber DFCs as functional ingredients. The incorporation of the DFCs into foods can be facilitated by their physicochemical properties. A significant concentration of bioaccessible phenolic compounds can be found after in vitro gastrointestinal digestion. We further observed a low concentration of phenolic compounds after in vitro colonic digestion of the DFCs, evidencing its chemical transformation by gut microbiota.

## Figures and Tables

**Table 1:** Proximate analysis of vegetable dietary fibre concentrates (DFC).

DFC	Moisture g/100 g fw	Protein g/100 g dw	Fat g/100 g dw	Ash g/100 g dw	Digestible Carbohydrate g/100 g dw	Dietary Fibre (DF) g/100 g dw
Artichoke	3.63 ± 0.32 ^d^	15.05 ± 0.32 ^b^	1.80 ± 0.13 ^b^	6.68 ± 0.15 ^a^	18.74 ± 0.91 ^d^	54.73 ± 0.36 ^a^
Red Pepper	10.16 ± 0.16 ^a^	12.17 ± 0.10 ^c^	4.77 ± 0.14 ^a^	4.73 ± 0.18 ^c^	35.43 ± 0.21 ^b^	42.90 ± 1.14 ^b^
Cucumber	8.15 ± 0.42 ^b^	18.52 ± 0.31 ^a^	1.65 ± 0.16 ^b^	7.12 ± 0.22 ^a^	39.01 ± 0.24 ^c^	33.70 ± 0.83 ^c^
Carrot	5.18 ± 0.22 ^c^	6.73 ± 0.23 ^d^	1.73 ± 0.18 ^b^	5.03 ± 0.12 ^b^	52.67 ± 0.23 ^a^	33.84 ± 1.60 ^c^

fw: fresh weight, dw: dry weight. Different lower-case letters in the same column indicate significant differences among DFC for each nutrient (*p* < 0.05).

**Table 2:** Physicochemical properties of vegetable dietary fibre concentrates (DFC).

DFC	pH	Acidity (g Acid/100 mL)	Apparent Density (g/mL)	Solubility (%)	Swelling Capacity (mL/g)	WHC (g Water/g)	ORC (g Oil/g)	WRC (g Water/g)	OHC (g Oil/g)
Artichoke	5.10 ± 0.01 ^a^	0.14 ± 0,01 ^c^	0.36 ± 0.01 ^d^	39.5 ± 0.6 ^a^	6.0 ± 0,1 ^c^	11.0 ± 0.6 ^c^	2.0 ± 0.1 ^a^	10.5 ± 0.2 ^b^	1.9 ± 0.1 ^a^
Red pepper	4.78 ± 0.01 ^b^	0.23 ± 0,01 ^b^	0.39 ± 0.01 ^b^	38.4 ± 0.2 ^a^	11.1 ± 0,1 ^a^	18.7 ± 0.6 ^a^	1.5 ± 0.1 ^b^	15.5 ± 0.2 ^a^	1.4 ± 0.1 ^b^
Cucumber	4.19 ± 0.01 ^d^	0.45 ± 0,03 ^a^	0.36 ± 0.01 ^d^	34.7 ± 1.5 ^b^	8.31 ± 0.1 ^b^	12.5 ± 0.9 ^b^	1.3 ± 0.1 ^bc^	9.8 ± 0.7 ^bd^	1.2 ± 0.1 ^c^
Carrot	4.25 ± 0.01 ^c^	0.16 ± 0,01 ^c^	0.47 ± 0.01 ^a^	26.4 ± 0.3 ^c^	8.04 ± 0.1 ^b^	9.4 ± 0.1 ^d^	1.2 ± 0.1 ^c^	9.4 ± 0.3 ^d^	1.1 ± 0.1 ^c^

Different lower-case letter in the same column indicate significant differences among DFC for each physicochemical property (*p* < 0.05).

**Table 3:** Phenolic compounds profile (mg/kg) of vegetable dietary fibre concentrate (DFC).

	ARTICHOKE DFC	RED PEPPER DFC	CUCUMBER DFC	CARROT DFC
PHENOLIC ACIDS				
Caffeic acid	551.5 ± 5.3	0.55 ± 0.06	nd	6.6 ± 0.4
Chlorogenic acid	1801.6 ± 34.8	2.8 ± 0.1	3.8 ± 0.2	178.4 ± 16.0
p-Coumaric acid	1.3 ± 0.1	2.8 ± 0.1	0.36 ± 0.06	0.07 ± 0.01
3,5 Dicaffeoyl quinic acid	5478.6 ± 46.4	0.41 ± 0.02	0.31 ± 0.03	3.7 ± 0.1
Ferulic acid	3.8 ± 0.2	10.9 ± 1.1	1.8 ± 0.1	1.5 ± 0.1
Gallic acid	13.7 ± 0.6	nd	nd	0.51 ± 0.03
Protocatechuic acid	320.5 ± 7.5	4.3 ± 0.4	0.50 ± 0.0	10.9 ± 1.1
p-Salicylic acid	2.48 ± 0.04	0.92 ± 0.03	11.3 ± 0.6	0.31 ± 0.02
o-Salicylic acid	7.34 ± 0.19	5.1 ± 0.2	3.6 ± 0.1	12.9 ± 0.4
Vanillic acid	4.7 ± 0.1	3.0 ± 0.0	0.34 ± 0.04	2.2 ± 0.4
Syringic acid	nd	0.57 ± 0.05	nd	nd
Total phenolic acids	8185.3	31.3	22.0	217.1
FLAVANONES				
Eriodictyol	1.6 ± 0.1	nd	nd	nd
Eriodictyol-7-O-rutinoside	1.3 ± 0.1	41.2 ± 2.4	0.46 ± 0.01	nd
Hesperitin-7-rutinoside	4.3 ± 0.2	104.9 ± 3.3	136 ± 21	0.11 ± 0.00
Naringenin 7-O-neohesperidoside	0.96 ± 0.04	2.1 ± 0.1	nd	nd
Naringenin-7-O-rutinoside	0.75 ± 0.03	1.9 ± 0.1	8.6 ± 0.1	nd
Total flavanone	8.9	149.9	145.6	0.11
FLAVONES				
Luteolin	38.4 ± 1.1	0.24 ± 0.02	nd	nd
Luteolin-7-O-glucoside	105.2 ± 5.5	1.3 ± 0.1	nd	0.17 ± 0.01
Luteolin-8-C-glucoside	nd	9.5 ± 0.1	nd	nd
Tangeretin	nd	nd	0.25 ± 0.01	nd
Apigenin-8-C-glucoside	nd	0.50 ± 0.14	0.43 ± 0.00	nd
Apigenin 6,8-di-C-glucoside	1.5 ± 0.1	9.4 ± 0.1	1.3 ± 0.1	nd
Total flavone	145.1	20.9	2.0	0.17
FLAVONOLS				
Quercetin	nd	2.1 ± 0.1	2.4 ± 0.2	nd
Quercetin-3-O-galactoside	0.18 ±0.00	nd	6.4 ± 0.4	nd
Quercetin-3-O-glucopyranoside	0.38 ± 0.01	1.8 ± 0.2	1.6 ± 0.5	nd
Quercetin-3-L-rhamnoside	0.16 ± 0.01	95.0 ± 4.7	5.4 ± 0.1	nd
Rutin	0.42 ± 0.01	0.82 ± 0.01	0.61 ± 0.02	nd
3-O-Methylquercetin 3-rutinoside	nd	1.3 ± 0.1	nd	nd
trans-Dihydroquercetin	0.25 ± 0.01	nd	nd	nd
Total flavonol	1.39	101.0	16.5	nd
OTHER PHENOLIC COMPOUNDS				
Isorhamnetin-3-O-glucoside	nd	0.90 ± 0.05	nd	nd
Phloridzin	nd	0.25 ± 0.01	9.6 ± 0.1	nd
TOTAL PHENOLIC COMPOUNDS	8340.7	304.4	195.7	217.4

nd: no detectable.

**Table 4:** Effect of gastrointestinal and colonic digestion on phenolic profile (mg/kg) of vegetable dietary fibre concentrate (DFC).

	ARTICHOKE DFC			RED PEPPER DFC			CUCUMBER DFC			CARROT DFC		
PHENOLIC ACIDS	IBF	INBF	CBF	IBF	INBF	CBF	IBF	INBF	CBF	IBF	INBF	CBF
Caffeic acid	1945.8 ± 85.3	263.5 ± 12.1	nd	5.8 ± 0.1	0.45 ± 0.1	nd	2.1 ± 0.2	1.9 ± 0.1	nd	213.0 ± 2.8	19.3 ± 0.6	nd
Chlorogenic acid	32,364.9 ± 55.01	6496.7 ± 14.6	20.6 ± 0.7	30.8 ± 0.5	5.2 ± 0.1	19.1 ± 1.0	16.6 ± 0.2	34.8 ± 0.3	12.9 ± 0.2	2155.6 ± 87.0	719.9 ± 19.6	30.1 ± 2.7
p-Coumaric acid	36.8 ± 0.3	3.3 ± 0.1	nd	15.5 ± 1.3	1.0 ± 0.1	nd	4.9 ± 0.1	1.9 ± 0.1	nd	8.6 ± 1.0	0.83 ± 0.04	nd
3,5 Dicaffeoyl quinic acid	5574.5 ± 49.4	1820.6 ± 3.13	5.4 ± 0.3	5.0 ± 0.1	1.2 ± 0.1	2.7 ± 0.3	nd	13.0 ± 0.1	nd	17.7 ± 0.3	17.6 ± 0.1	2.5 ± 0.4
Ferulic acid	22.5 ± 2.3	3.0 ± 0.2	nd	58.7 ± 3.5	1.1 ± 0.1	nd	3.4 ± 1.9	4.7 ± 0.1	2.9 ± 0.3	27.7 ± 1.0	3.7 ± 0.1	nd
Gallic acid	nd	4.3 ± 0.1	nd	nd	nd	nd	nd	nd	nd	nd	nd	nd
Protocatechuic acid	2120.6 ± 46.1	712.5 ± 29.8	3.2 ± 0.3	63.4 ± 6.7	1.7 ± 0.1	nd	13.4 ± 0.7	4.7 ± 0.5	nd	413.0 ± 1.5	24.7 ± 0.2	2.4 ± 0.1
p-Salicylic acid	nd	0.57 ± 0.01	nd	nd	0.72 ± 0.1	nd	nd	0.57 ± 0.04	nd	nd	0.57 ± 0.01	nd
o-Salicylic acid	nd	2.0 ± 0.1	52.2 ± 1.4	nd	1.0 ± 0.1	28.9 ± 1.2	nd	1.2 ± 0.1	19.4 ± 0.4	nd	3.5 ± 1.3	44.6 ± 2.4
Vanillic acid	7.4 ± 0.1	2.8 ± 0.1	3.7 ± 0.1	8.2 ± 0.3	0.66 ± 0. 06	3.1 ± 0.5	2.4 ± 0.1	2.9 ± 0.1	7.7 ± 0.3	nd	1.4 ± 0.01	2.2 ± 0.2
Syringic acid	nd	nd	22.9 ±0.7	1.5 ± 0.1	nd	4.2 ± 0.4	nd	0.58 ± 0.03	4.8 ± 0.1	nd	nd	nd
Total phenolic acids	42,072.5	9309.4	85.3	189.2	13.1	58.1	40.7	63.9	42.8	2835.5	791.4	81.9
FLAVANONE												
Eriodictyol	3.7 ± 0.1	3.9 ± 0.4	nd	nd	nd	nd	nd	nd	nd	nd	nd	nd
Eriodictyol-7-O-rutinoside	4.4 ± 0.1	2.4 ± 0.1	nd	40.6 ± 0.3	1.3 ± 0.1	nd	nd	18.3 ± 0.6	nd	nd	nd	nd
Hesperitin-7-rutinoside	15.5 ± 1.3	17.2 ± 1.0	nd	121.2 ± 1.1	204.0 ± 6.4	nd	346.7 ± 4.6	96.5 ± 1.8	nd	3.7 ± 0.1	1.4 ± 0.1	nd
Naringenin-7-O-neohesperidoside	1.6 ± 0.1	0.84 ± 0.07	nd	2.2 ± 0.2	nd	nd	nd	1.6 ± 0.1	nd	nd	nd	nd
Naringenin-7-O-rutinoside	8.5 ± 0.4	nd	nd	4.5 ± 0.1	nd	nd	28.5 ± 2.3	nd	nd	nd	nd	nd
Total flavanone	33.6	24.4		168.6	205.3		375.2	116.4	nd	3.7	1.4	
FLAVONE												
Luteolin	nd	182.7 ± 3.0	nd	nd	nd	nd	nd	nd	nd	nd	nd	nd
Luteolin-7-O-glucoside	nd	158.7 ± 3.7	nd	nd	nd	nd	nd	2.5 ± 0.1	nd	nd	2.3 ± 0.2	nd
Luteolin-8-C-glucoside	nd	nd	nd	23.2 ± 1.6	nd	nd	nd	8.3 ± 0.7	nd	nd	nd	nd
Tangeretin	nd	nd	nd	nd	nd	nd	nd	nd	nd	nd	nd	nd
Apigenin 6,8-di-C-glucoside	6.0 ± 0.3	nd	nd	28.8 ± 2.2	2.0	nd	5.5 ± 0.3	6.6 ± 0.3	nd	nd	nd	nd
Total flavone	6.0	341.4	nd	52.1	2.0		5.5	15.0	nd		2.3	
FLAVONOL												
Quercetin	nd	nd	nd	78.2 ± 0.3	3.6 ± 0.2	nd	nd	61.0 ± 1.0	nd	nd	nd	nd
Quercetin-3-O-galactoside	nd	0.76 ± 0.1	nd	nd	nd	nd	4.7 ± 0.2	2.2 ± 0.1	nd	nd	nd	nd
Quercetin-3-O-glucopyranoside	nd	1.1 ± 0.1	nd	4.4 ± 0.24	2.4 ± 0.1	nd	1.5 ± 0.2	2.7 ± 0.1	nd	nd	nd	nd
Quercetin-3-L-rhamnoside	nd	nd	nd	157.4 ± 12.2	6.0 ± 0.3	nd	5.6 ± 0.6	108.1 ± 6.5	nd	nd	nd	nd
Rutin	2.0 ± 0.1	1.7 ± 0.1	nd	1.8 ± 0.2	1.5 ± 0.1	nd	1.4 ± 0.1	1.3 ± 0.1	nd	nd	nd	nd
3-O-Methylquercetin 3-rutinoside	nd	nd	nd	3.5 ± 0.2	nd	nd	nd	1.7 ± 0.2	nd	nd	nd	nd
Total flavonol	2.0	3.5		245.3	13.5		13.2	176.9	nd	nd	nd	nd
OTHER PHENOLIC COMPOUNDS												
Isorhamnetin-3-O-glucoside	nd	nd	nd	1.6 ± 0.1	nd	nd	nd	nd	nd	nd	nd	nd
Phloridzin	nd	nd	nd	15.5 ± 0.8	6.7 ± 0.5	2.7 ± 0.2	16.3 ± 1.4	1.1 ± 0.1	nd	nd	nd	nd
TOTAL PHENOLIC COMPOUNDS	42,114.2	9678.7	85.3	672.3	240.6	60.8	451.0	373.4	42.8	2839.2	795.2	81.9

IBF: Intestinal Bioaccessible fraction; INBF: Intestinal non-bioaccessible fraction; CBF: Colon bioaccessible fraction, nd: no detectable.

## Data Availability

The data used to support the findings of this study can be made available by the corresponding author upon request.
